# Parental Hesitancy and Concerns around Accessing Paediatric Unscheduled Healthcare during COVID-19: A Cross-Sectional Survey

**DOI:** 10.3390/ijerph17249264

**Published:** 2020-12-11

**Authors:** Emma Nicholson, Thérèse McDonnell, Ciara Conlon, Michael Barrett, Fergal Cummins, Conor Hensey, Eilish McAuliffe

**Affiliations:** 1 UCD Centre for Interdisciplinary Research Education and Innovation in Health Systems, UCD School of Nursing, Midwifery & Health Systems, University College Dublin, D04 V1W8 Dublin, Ireland; therese.mcdonnell@ucd.ie (T.M.); ciara.conlon@ucd.ie (C.C.); Eilish.McAuliffe@ucd.ie (E.M.); 2 Children’s Health Ireland at Crumlin, D12 N512 Dublin, Ireland; Michael.Barrett@olchc.ie; 3 Women’s and Children’s Health, School of Medicine, University College Dublin, D04 V1W8 Dublin, Ireland; 4 National Children’s Research Centre, D12 N512 Dublin, Ireland; 5 REDSPOT, Emergency Department, Limerick University Hospital, V94 F858 Limerick, Ireland; Fergal.Cummins@hse.ie; 6 Children’s Health Ireland at Temple Street, D01 XD99 Dublin, Ireland; Conor.hensey@cuh.ie

**Keywords:** COVID-19, cross-sectional survey, parents, paediatric healthcare, hesitancy, avoidance

## Abstract

A decrease in attendance at emergency departments among paediatric populations has been reported during the Coronavirus Disease 2019 (COVID-19) pandemic. The present study sought to understand parents’ hesitancy and concerns around accessing healthcare during the pandemic using a cross-sectional survey of parents of children under the age of 16 (*N* = 1044) in Ireland. Multinomial and logistic regression analyses were used to determine the factors that influenced avoidance and hesitancy. In total, 34% of participants stated that their child required healthcare during the pandemic, of whom 22% decided against seeking healthcare. Parents who reported being much more hesitant about accessing healthcare were more likely to report mild–moderate (Relative Risk Ratio (RRR) = 2.31, CI: 1.54–3.47) and severe–extremely severe stress (RRR: 3.37, CI: 1.81–6.27). Parents who understood government advice to mean avoiding health services were more likely to be hesitant to attend (RRR: 1.71, CI; 1.10–2.67). These effects held when restrictions were beginning to be lifted. Higher levels of stress were associated with a parent believing that the government advice meant that they should not attend health services (OR: 1.66, CI: 1.14–2.41). Public health messaging must ensure parents are reassured on the accessibility and safety of paediatric healthcare services as this public health emergency continues.

## 1. Introduction

The most effective public health response to delay and reduce the spread of COVID-19 appears to be widespread restrictions on movement and physical interaction [1]. These restrictions include the closure of schools, sporting, social and cultural activities and institutions, banning of large gatherings of people, and the closure of non-essential workplaces. In many countries, such public health measures have never before been implemented at the national level, and therefore the impacts of the measures on behaviours such as avoidance of health services are unknown. The public health response to delay the spread of SARS in Canada resulted in unwarranted avoidance behaviour in areas of low incidence, and it has been suggested that more appropriate public health advice may have reduced such avoidance behaviour [2]. Moreover, during the H1N1 outbreak in Hong Kong, approximately two-thirds of people avoided going to hospital [3], and having small children was also found to increase avoidance behaviours during the H1N1 epidemic in a Turkish population [4]. A hypothetical study examining public perception of risk during an influenza pandemic found that approximately 40% of respondents stated that they would avoid hospital for fear of infection [5].

Understanding the behaviour of populations during a pandemic is key to ensuring the maintenance of health for the duration of the public health emergency and to inform future communication strategies [6]. Hospital avoidance during the COVID-19 pandemic has been reported with a significant decrease in attendance at emergency departments (ED) among paediatric populations, with potential increased morbidity and mortality outcomes [7,8,9]. While this decrease can be partially explained by fewer social interactions resulting in a lower risk of contracting some infectious diseases, as well as a reduction in minor accidents and injuries, it is also likely the result of a shift in the health-seeking behaviour of parents [10,11]. Of particular concern are children with pre-existing conditions, who may be at increased risk due to the impact of delays in seeking healthcare [7,12]. While evidence of healthcare avoidance during COVID-19 is apparent, less is known about the profiles of parents and children who may be more hesitant to access healthcare during the pandemic.

Individual behaviours during public health emergencies will vary based on personal characteristics such as perception of risk, personal circumstances, and personality [4,13], however public health messaging can play a vital role in the behavioural responses of the population. Public responses to epidemics such as SARS or H1NI have been found to vary cross-culturally and are influenced by factors such as previous experiences of epidemics, structuring of health systems, and national public health communication strategies [3]. Broader impacts of the pandemic and restrictions may include worsening stress levels and mental health for parents [14], which may exacerbate avoidance behaviours and concerns around their child’s health. The impacts of broad national public health restrictions on stress and avoidance behaviours for COVID-19 remain unclear.

The aim of the present study was to examine avoidance behaviour and the level of hesitancy in parents towards accessing healthcare for their child during the COVID-19 pandemic and to determine the factors associated with healthcare avoidance and hesitancy.

## 2. Materials and Method

### 2.1. Survey Design

**Figure 1:**
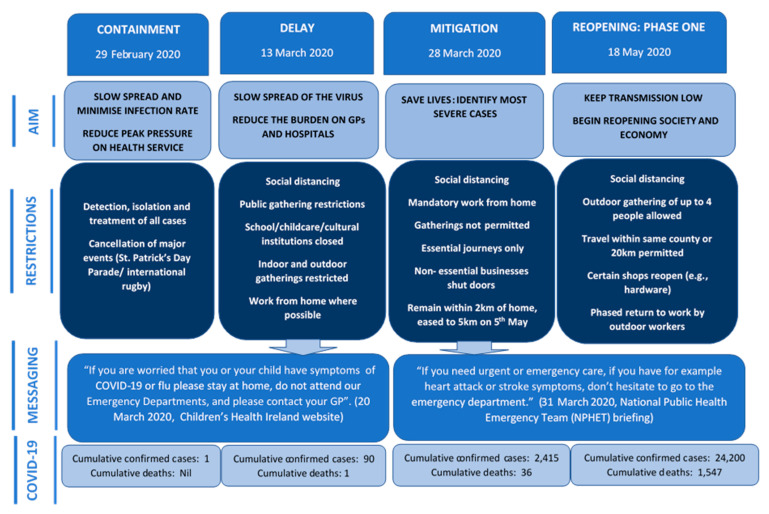
Public health stages of COVID-19 response.

A cross-sectional survey was designed by the multi-disciplinary research team to capture the experience of parents accessing healthcare for their child during the COVID-19 pandemic. Face validity was sought from a sample of parents in Ireland to ensure that the questions were clear and relevant to the target population, and from frontline staff to ensure it was relevant to their experience of delivering healthcare during the pandemic. The survey collected demographic information to profile parents and children and capture relevant health information, such as pre-existing conditions, chronic illness or disability, and healthcare entitlement. In Ireland, approximately 33% of the population qualify for free access to primary and hospital care under the General Medical Service (GMS) scheme available to those on low incomes or with a specified illness, a further 10% have access to free care by a general practitioner (GP only visit card) based on income or age [15], with the remainder paying an average of €51 per visit to their GP [16] or an out-of-hours service and €100 for access to a public ED. The incidences of COVID-19 and self-isolation were assessed, as well as respondents’ concerns about their child contracting COVID-19 and their use or avoidance of healthcare for their child during the “lockdown”. Hesitancy in accessing healthcare services at two distinct stages of the public health response was also assessed; firstly as the country entered the initial “*delay and mitigation*” phases, or “lockdown” as it was known within public discourse, when schooling, business, and travel restrictions were put in place (beginning on the 12 March 2020); and secondly during “*phase one*” of reopening, the first stage of official easing of restrictions (commencing 18 May 2020), the stage at which the survey was conducted. Figure 1 [9] provides a detailed outline of the public health stages of the COVID-19 response in Ireland. The survey was delivered online through Qualtrics™ and is available as Appendix A.

Stress was measured using the stress subscale from the Depression, Anxiety, and Stress Scale (DASS-21) [17]. This subscale has been shown to have excellent psychometric properties across different cultural settings [18]. It has acceptable internal consistency (Cronbach’s alpha = 0.78–0.86) and very good construct validity against related measures such as satisfaction, social support, and resilience [18].

### 2.2. Sampling and Data Collection

Participants were recruited using Qualtrics™ market research panels, which collect data from a diverse network of respondents across Ireland in partnership with the European Society for Opinion and Marketing Research (ESOMAR). Participants received a small incentive for taking part in the survey. Based on recruitment procedures, approximately 1100 participants were targeted by Qualtrics™ via email to take part in the study, resulting in a satisfactory response rate of 95%. The survey was administered online through Qualtrics™ software by Qualtrics™ and took approximately 10 min to complete. Respondents were randomly selected from the panels once they met the inclusion criteria. The inclusion criteria for the study were parents of children under the age of 16 in Ireland. In order to ensure an adequate representation of all age groups, at least 25% of participants were required to have children from ages 0 to 4, 25% from ages 5 to 9, and 25% from ages 10 to 16. The data underwent quality checks with any participant who responded in a particular pattern, while those who responded under a set time limit removed from the final sample. Data was collected from 5 June 2020 to 10 June 2020 during phase one of easing of restrictions.

### 2.3. Data and Data Analysis

The sample size (*N* = 1044) was selected to provide an adequate sample to conduct the analysis. Descriptive analysis was used to review the demographic characteristics of the sample, personal experience of COVID-19, concerns held by participants, sources of information, the prevalence of healthcare avoidance by parents during the pandemic, and levels of hesitancy at two timepoints (the “delay and mitigation” phase and phase one of reopening).

Multinomial and logistic regression was used to establish the factors that predicted reported hesitancy and avoidance behaviour, respectively, and to control for any potential confounding variables. Some categorical variables were restated as binary to ensure adequate sample size and to aid interpretation, such as age of parent (under 40/over 40), education (below degree/degree or post graduate), concern around child contracting COVID-19 (slightly concerned or not at all/highly or moderately concerned), concern about the effect of COVID-19 on the child (somewhat unwell or unaffected/very or quite unwell), and ease of access to the general practitioner or ED (very or usually easy/can if I have to, somewhat or very difficult). For the DASS stress categories, mild and moderate stress were collapsed into one category and severe and extremely severe stress were collapsed into a second category. The analyses were carried out using STATA 15.0.

### 2.4. Ethics Approval and Consent of Participants

The study was granted full ethical approval by the COVID-19 National Research Ethics Committee (NREC) in Ireland (ref: 20-NREC-COV-034). All participants provided informed consent online prior to participating in the survey.

## 3. Results

### 3.1. Participants

Parents of children under the age of 16 living in Ireland (*N* = 1044) were recruited to the study, of which 62% were female and 83.7% were either married or cohabiting. Here, 52.7% had a degree or postgraduate qualification, which is reflective of the national average for those aged 25–64 [19]. Furthermore, 53.1% had a child or children aged four or under, 52% had children aged 10 to 16, and 60.4% had more than one child. A comprehensive geographical spread was achieved, with each county of Ireland represented. Here, 46% of participants reported that a person in their household had an underlying health condition, 34% of whom were children under 16. The number of respondents who held a GMS card was 38%, which is slightly higher than the national proportion in 2018 (33%) [15], however the proportion of private health insurance holders accurately reflects the current national average (46.2%) [20]. Overall, the sample was a good representation of the general population on important factors for health seeking behaviour such as medical card status and education [21]. Demographic information pertaining to participants can be found in Table 1.

### 3.2. Descriptive Analysis

#### 3.2.1. Accessing Healthcare during COVID-19

Here, 34% of participants stated that their child required healthcare during the pandemic, of whom 22% (*n* = 80) decided against seeking healthcare. Of those that did, 42% had a face-to face consultation with a general practitioner (GP) or through private facilities, while 39% had a remote consultation by phone or video. Furthermore, 13% of those that accessed healthcare did so at an emergency department (ED).

#### 3.2.2. Experience of COVID-19 and Concerns about Contracting COVID-19

Here, 1.3% of participants (*n* = 11) indicated they or another household member had a confirmed case of COVID-19, while 25% of participants stated they or another household member self-isolated due to COVID-19. Almost half of participants were slightly concerned (46%) about the risk of their child contracting COVID-19, 32% were moderately concerned, while 9% felt their child was at high risk. Furthermore, 50% said their child would be somewhat affected if they contracted COVID-19, while 28% said they would be quite unwell and 10% thought their child would be very unwell.

#### 3.2.3. Sources of Information and Concerns about Accessing Healthcare

Participants were asked where they sought information on accessing healthcare for their child following the onset of the pandemic. The majority (88.5%) utilized either official government or Health Service Executive (HSE) sources. Here, 18% accessed information through friends and family on social media, while 17% turned to “experts” on social media.

Participants were asked to select the reasons that caused the greatest amount of concern when considering accessing healthcare for their child during COVID-19. Participants could select more than one reason. Fear of contracting COVID-19 (67.6%, *n* = 706) was the most commonly reported concern, while 30.2% (*n* = 315) stated that they were concerned the service would be busy at this time. Furthermore, 25.2% (*n* = 263) believed that services were needed more by others, while 24.4% (*n* = 255) feared they would be judged by healthcare professionals for attending. In total, 17.5% (*n* = 183) reported that they thought the public health advice issued by the government was to avoid health services. Additionally, 9.1% (*n* = 95) reported travel concerns such as a lack of access to a car and not wanting to use public transport, while 8.8% (92) reported another reason.

#### 3.2.4. Hesitancy Regarding Access of Healthcare

Participants were asked their level of hesitancy about accessing healthcare, both during the delay and mitigation or “lockdown” stage and during phase one of reopening, by selecting one of five points on a scale ranging from much more to much less. In total, 23% stated that they were much more hesitant and 35% were somewhat more hesitant during delay and mitigation, however this reduced to 5% and 18% respectively during phase one. Furthermore, 34% felt about the same regarding accessing healthcare during delay and mitigation, increasing to 47% during phase one. Additionally, 19% of participants indicated they were hesitant at both stages.

#### 3.2.5. Stress

Here, 65% of the sample (*n* = 679) fell within the normal range on the stress subscale of the DASS, while 12.7% (*n* = 133) and 13.2% (*n* = 138) reported mild or moderate stress, respectively. Additionally, 7.2% (*n* = 75) of the sample fell within the severe range, while 1.8% (*n* = 19) of participants were extremely severe.

### 3.3. Regression Analyses

#### 3.3.1. Hesitancy to Attend Health Services

The results from multinomial regression analyses estimating the association between reported hesitancy to attend health services and demographic-, health-, and COVID-related characteristics, at both the delay and mitigation phase and phase one of reopening, are represented in Table 2.

During the delay and mitigation phase, females were twice as likely to report being much more hesitant (RRR = 1.94, CI: 1.33–2.82). Higher than normal stress levels were also significantly associated with hesitancy when compared to respondents with normal stress levels. Those who reported being much more hesitant were over twice as likely to report mild–moderate stress (RRR = 2.31, CI: 1.54–3.47), while those with severe–extremely severe stress were over three times more likely (RRR: 3.37, CI: 1.81–6.27). Finally, those who felt that the government advice was to stay away from health services were 1.7 times more likely to be much more hesitant (RRR: 1.71, CI: 1.10–2.67).

In phase one of reopening, respondents who reported having severe or extremely severe stress levels were over five times more likely to be much more hesitant (RRR: 5.22, CI: 2.22–12.29), while those with mild or moderate stress levels were over three times more likely (RRR: 3.10, CI: 1.56–6.20) compared with those with normal levels of stress. Similarly, there was a positive association between being much more hesitant in phase one and believing the government advice was to stay away (RRR: 2.19, CI: 1.12–4.29). Parents who accessed healthcare for their child during the delay and mitigation phase were no more or less hesitant during phase one than parents who had no experience of accessing healthcare for their child since the onset of the pandemic.

#### 3.3.2. Believed the Official Public Health Advice Was to Avoid Health Services

Logistic regressions were carried out to examine the factors that contributed to the understanding that government advice was to stay away from health services. The results are reported in Table 3.

When using the full sample (*N* = 1044), those who reported mild or moderate stress levels were 1.7 times more likely to believe that the government advice meant that they should not attend health services (OR: 1.66, CI: 1.14–2.41), while those with severe or extremely severe stress were nearly twice as likely (OR: 1.87, CI: 1.10–3.20) as those with normal stress levels. Looking specifically at the group who needed to access healthcare during the lockdown (*n* = 360), those with mild or moderate stress levels were 2.4 times more likely to have reported that they thought government advice was to stay away (OR: 2.36, CI: 1.12–4.99). Those that reported severe or extremely severe stress were also more likely to hold this perception, however this was not significant (OR: 2.29, CI: 0.87–6.07). Parents who perceived their child to be at high or moderate risk of contracting COVID-19 were 3.4 times more likely to believe the official public advice was to avoid healthcare services (OR: 3.40, CI: 1.59–7.28). On balance, those that were very or quite concerned about the effect COVID-19 would have on their child were less likely to hold this view (OR: 0.43, CI: 0.20–0.94).

## 4. Discussion

This study sought to assess the factors that influence parents when seeking healthcare for their children during the implementation of public health measures and restrictions to delay the spread of COVID-19. Delayed presentation or avoidance of the emergency department during the COVID-19 pandemic has been a cause for concern [11,12], however there is little understanding of the characteristics of those that have avoided or would be hesitant to attend health services. Approximately one-third of the present sample required healthcare for their children during the public health restrictions to delay the spread of COVID-19, but one-fifth of these parents avoided accessing such healthcare when needed. Of those that required healthcare, parents who avoided were more likely to report that the services were needed more by others. The survey also assessed the degree to which parents were hesitant to attend health services for their children during both the most severe and limiting restrictions (i.e., delay and mitigation period) and during the first step in the plan to ease the restrictions (i.e., phase one). Factors that influenced the extent to which parents were hesitant included higher than normal levels of stress and their concern around their child contracting COVID-19. Moreover, the misinterpretation of the government’s public health advice also played a role in whether parents were more likely to be hesitant.

While there was significant reliance on official government advice, a key finding in the study was that a proportion of the sample may have misunderstood this government advice, which may have resulted in unnecessary avoidance of or hesitancy to access health services. The unprecedented nature of this global pandemic has resulted in the release of a substantial amount of information into the public domain, which has necessitated the need for clear and tailored public health messaging from health authorities and government bodies [22]. Nevertheless, even clear and coherent messaging around the importance of hand-washing and maintaining physical distancing, coupled with the concern of contracting COVID-19, requires the ability to obtain, understand, and use this information to make informed decisions [23]. Such health literacy is vital in the maintenance of public health, however social determinants of health literacy can significantly disadvantage particular sections of the population [24] and exacerbate existing health inequalities [25], which future research could examine in the context of the COVID-19 pandemic. Moreover, economic uncertainty and social isolation may increase anxiety and stress [25]. In the present study, stress was found to be a significant driver of hesitancy and misunderstanding of public health advice. Public health officials need to be cognizant of the unintended consequences of the messages designed to inform the public as the pandemic progresses and during future public health emergencies.

Understanding the public’s concerns and perceptions of the risk of contracting COVID-19 is important for the effectiveness and success of government responses to control the disease [26]. In the current study, a greater concern that their child would contract COVID-19 was linked to hesitancy to attend health services. Public compliance is required for wide-ranging public health strategies to be effective, such as physical distancing, hand hygiene, and closure of schools and non-essential workplaces [1,26]. The use of formal information sources are critical for encouraging protective behaviours such as social distancing [27]. However, it is important for public health messaging and health services to adequately communicate to the public that essential services such as healthcare facilities remain open and are safe to attend when needed to avoid deterioration of a child’s illness [7]. The decision to seek healthcare is complex [21] and current circumstances require parents to weigh up information regarding their child’s need for healthcare for a non-COVID-19 related illness, the risk of their child catching COVID-19, and the government public heath advice. Moreover, experience of COVID-19 may also impact their perception of this risk [26], however only a small number of the current participants had direct personal experience of contracting or self-isolating due to the disease. It is worth noting that a commonly reported reason for the drastic public measures was the need to maintain capacity in health services for the most vulnerable members of the population [23], however this may be less relevant to paediatric-specific services given that children were less affected by COVID-19. This messaging may explain why many parents in the present survey stated that they felt that services were needed more by others or that they did not want to burden the health system at such a time. Emotional engagement with public health messages can also elicit unknown responses and needs to be better understood in the context of COVID-19 [28]. Indeed, fear of COVID-19 was a key cause for concern in the present sample. Public health messaging tends to be expert-driven, yet an approach which engages with target communities may reduce the likelihood of misinterpretation and any potential unintended consequences that may occur as a result of public health messaging [29].

### Limitations

It was not possible to determine whether the sample population recruited in the present study was representative of the general population. However, the sample population largely aligns with national figures on factors such as education and health insurance status, and there was an adequate spread across age groups and geographical regions. Females were over-represented in the sample, however this is common in studies of parental behaviour. There was no information on those who declined to take part in the study. Data collection occurred during the first step of lifting restrictions, and therefore reporting on hesitancy during the delay and mitigation phase was retrospective and may be subject to recall bias. As specific information on the clinical reason for requiring healthcare was not collected, we have no information on the severity of the illness for those who accessed or avoided healthcare. However, understanding when and why patients seek healthcare is highly complex and requires in-depth knowledge of how individuals perceive their health and health services [30]; thus, such information may not have provided more context to the present results. Finally, for the purposes of the current study, unscheduled health services were conceptualized as one system, because previous evidence suggests that patients view such services as a single system as opposed to separate services with distinct boundaries [21,30,31]. However, given the changes to service provision during the pandemic, features of certain health services such as emergency departments may elicit more anxiety than others due to contextual factors.

## 5. Conclusions

The present study sought to understand parents use of health services and their concerns around accessing health services during the COVID-19 pandemic and aimed to provide context as to why some parents may have avoided or were hesitant to access healthcare during this time. Misinterpretation of government public health messaging and stress appeared to contribute to parental avoidance of or hesitancy to utilize healthcare services during the public health measures imposed to combat COVID-19. Concern around their child contracting COVID-19 was related to an increased likelihood of believing the government advice was to stay away from health services. The longer-term impact of such avoidance remains unknown with regards to clinical outcomes and mortality for non-COVID-19 conditions.

## Figures and Tables

**Table 1:** Sample characteristics.

*N* = 1044		Total % (*n*)	
Gender of Parent	Female	62.1 (648)	
**Age of Parent**	<29	12.0 (125)	
	30–39	41.2 (430)	
	40–49	37.2 (388)	
	>50	9.7(101)	
**Marital Status**	Single	10.6 (111)	
	Married	68.2 (712)	
	Cohabiting	15.5 (162)	
	Divorced/Separated/Widowed	5.7 (59)	
**Location**	Dublin	27.2 (284)	
	Cork	10.8 (113)	
	Galway	6.9 (72)	
	Rest of country	55.1 (575)	
**Age of Children**	<2	23.1 (241)	
	2–4	30.0 (313)	
	5–9	45.9 (479)	
	10–16	52.0 (543)	
**No. of Children**	One child	39.6 (413)	
	Two children	38.9 (406)	
	Three or more children	21.6 (225)	
**Education**	Lower Secondary	4.5 (47)	
	Upper Secondary	14.0 (146)	
	Post-Secondary certificate/vocational	28.8 (301)	
	Degree or Postgraduate	52.7 (550)	
**Health Cover Status**	No cover	18.5 (193)	
	Medical card only	27.8 (290)	
	GP visit card only	8.1 (84)	
	Private insurance only	35.5 (371)	
	Both insurance and medical card	10.2 (106)	
**Profession**	Healthcare professional	10.6 (111)	
**Underlying Health Condition**	Underlying health conditions in household	46.4 (484)	
	Child under 16 with underlying health condition	15.8 (165)	
	Other household member with underlying health condition	37.9 (396)	
		Child under 16	Other household member
**Type of condition**	Cancer	0.6 (6)	3.5 (37)
	Chronic lung condition, e.g., Asthma	9.0 (94)	11.8 (123)
	Diabetes	1.2 (12)	7.9 (82)
	High blood pressure/Hypertension	0.4 (4)	18.4 (192)
	Immunocompromising condition	2.3 (24)	12.2 (127)
	Intellectual disability	4.9 (51)	2.2 (23)
	Heart/kidney/liver disease or organ transplant	1.2 (12)	2.9 (30)

**Table 2:** Multinomial regression results.

Reporting Hesitancy in Both “Delay and Mitigation” and “Phase One” Periods and 95% CI.						
Relative Risk Ratio (RRR) *N* = 1044	Delay and Mitigation Period (“Lockdown”)			Phase One of Reopening		
*Ref: About the same*	Much more (*N* = 242)	Somewhat more (*N* = 366)	Somewhat or much less (*N* = 79)	Much more (*N* = 54)	Somewhat more (*N* = 184)	Somewhat or much less (*N* = 317)
Gender: Female	1.94 ** (1.33–2.82)	1.37 * (1.00–1.88)	0.71 (0.43–1.20)	1.33 (0.71–2.48)	1.16 (0.80–1.67)	1.67 ** (1.23–2.28)
Participant Age: Under 40	0.90 (0.63–1.29)	1.01 (0.74–1.38)	1.53 (0.89–2.61)	0.79 (0.41–1.50)	1.25 (0.86–1.82)	0.82 (0.61–1.12)
Marital Status: Married or cohabiting	0.95 (0.59–1.52)	1.38 (0.90–2.13)	1.57 (0.75–3.30)	0.60 (0.27–1.32)	1.06 (0.64–1.77)	0.77 (0.51–1.16)
Education: Degree or Post-graduate	1.14 (0.79–1.65)	1.17 (0.86–1.61)	0.95 (0.56–1.63)	0.90 (0.48–1.68)	0.98 (0.67–1.41)	0.87 (0.64–1.19)
Health Cover (ref: no cover)						
*GMS card only*	0.86 (0.50–1.46)	0.99 (0.63–1.55)	2.52 * (1.14–5.57)	1.00 (0.35–2.88)	0.90 (0.52–1.54)	0.64 * (0.41–0.99)
*GP visit card only*	1.70 (0.81–3.57)	1.65 (0.87–3.14)	2.52 (0.87–7.28)	0.73 (0.14–3.90)	1.14 (0.56–2.33)	0.70 (0.38–1.31)
*Health insurance*	1.29 (0.79–2.13)	1.20 (0.79–1.84)	1.11 (0.48–2.60)	1.95 (0.73–5.15)	1.06 (0.63–1.79)	1.09 (0.73–1.65)
*Health insurance and Medical card*	1.22 (0.60–2.50)	1.42 (0.77–2.63)	2.62 (0.93–7.32)	3.14 (0.98–10.10)	1.27 (0.64–2.55)	0.83 (0.45–1.54)
Healthcare worker	0.87 (0.48–1.58)	0.90 (0.54–1.49)	1.23 (0.55–2.75)	2.15 (0.89–5.20)	1.15 (0.65–2.03)	0.83 (0.50–1.38)
No. of Children (ref: three children or more)						
*One child*	0.72 (0.44–1.17)	0.49 ** (0.32–0.75)	0.33 ** (0.17–0.65)	1.04 (0.45–2.40)	0.89 (0.55–1.43)	0.57 ** (0.38–0.85)
*Two children*	0.85 (0.52–1.39)	0.64 * (0.42–0.98)	0.56 (0.30–1.06)	1.09 (0.47–2.53)	0.91 (0.57–1.47)	0.78 (0.53–1.14)
Health condition household	1.08 (0.72–1.63)	0.99 (0.70–1.41)	1.09 (0.61–1.95)	0.63 (0.31–1.28)	1.07 (0.71–1.61)	1.22 (0.87–1.72)
Health condition under 16 child	1.45 (0.83–2.51)	1.34 (0.81–2.22)	0.75 (0.32–1.79)	0.36 (0.11–1.11)	0.76 (0.44–1.32)	0.77 (0.48–1.23)
DASS Stress (ref: normal)						
*Mild/moderate*	2.31 *** (1.54–3.47)	1.46 * (1.01–2.10)	1.03 (0.55–1.92)	3.10 ** (1.56–6.20)	1.91 ** (1.27–2.87)	1.34 (0.94–1.91)
*Severe/extremely severe*	3.37 *** (1.81–6.27)	1.48 (0.79–2.77)	1.18 (0.43–3.22)	5.22 *** (2.22–12.29)	1.80 (0.98–3.30)	1.12 (0.63–1.99)
Very/quite concerned effect COVID-19 on child	1.45 (0.98–2.13)	0.91 (0.65–1.29)	1.09 (0.63–1.90)	1.44 (0.74–2.79)	1.04 (0.70–1.53)	0.68 * (0.49–0.96)
High risk/moderately concerned risk contracting COVID-19 child	1.85 ** (1.27–2.70)	1.22 (0.87–1.71)	1.87* (1.09–3.22)	2.78 ** (1.43–5.40)	1.87 ** (1.28–2.73)	1.24 (0.90–1.72)
Gov advice stay away	1.71 * (1.10–2.67)	1.17 (0.77–1.78)	0.75 (0.35–1.59)	2.19* (1.12–4.29)	1.19 (0.76–1.87)	1.01 (0.68–1.51)
Easy access to GP	1.15 (0.77–1.72)	1.09 (0.77–1.54)	0.80 (0.46–1.41)	1.51 (0.74–3.08)	1.30 (0.87–1.95)	1.09 (0.79–1.52)
Easy access to ED	1.09 (0.74–1.60)	0.97 (0.70–1.36)	0.91 (0.51–1.60)	1.18 (0.61–2.29)	1.11 (0.76–1.64)	0.94 (0.68–1.29)
Household member self-isolated	1.17 (0.77–1.79)	1.24 (0.85–1.81)	0.70 (0.36–1.36)	1.13 (0.55–2.34)	1.10 (0.72–1.69)	1.00 (0.70–1.45)
Accessed healthcare	n/a	n/a	n/a	0.96 (0.46–2.00)	1.24 (0.82–1.87)	1.24 (0.87–1.76)

Note: 95% confidence intervals in parentheses; * *p* < 0.05, ** *p* < 0.01, *** *p* < 0.001.

**Table 3:** Logistic regression results for participants who believed government advice was to stay away from health services.

Odds Ratio (95% CI)	Full Sample (*n* = 1044)	Sample That Required Healthcare (*n* = 360)
Gender: Female	0.96 (0.69–1.36)	1.43 (0.69–2.96)
Participant Age: Under 40	1.18 (0.83–1.66)	1.83 (0.84–3.97)
Marital Status: Married or cohabiting	1.45 (0.89–2.37)	0.94 (0.36–2.48)
Education: Degree or Postgraduate	1.39 (0.98–1.97)	1.50 (0.73–3.09)
Health cover		
GMS card only	1.24 (0.74–2.06)	1.95 (0.55–6.83)
GP visit card only	1.11 (0.55–2.25)	2.00 (0.40–9.91)
Health insurance	1.05 (0.64–1.71)	1.52 (0.42–5.46)
Health insurance and Medical card	1.32 (0.69–2.52)	3.13 (0.83–11.82)
Healthcare worker	0.64 (0.36–1.16)	0.83 (0.32–2.12)
No. of Children: (ref: three children or more)		
One child	1.37 (0.87–2.16)	1.14 (0.48–2.68)
Two children	1.08 (0.68–1.71)	0.97 (0.43–2.19)
Health condition household	1.10 (0.75–1.60)	2.69 * (1.21–5.98)
Health condition under 16 child(ren)	0.86 (0.51–1.46)	0.85 (0.37–1.95)
DASS Stress: (ref: normal)		
Mild/moderate	1.66 ** (1.14–2.41)	2.36 * (1.12–4.99)
Severe/extremely severe	1.87 * (1.10–3.20)	2.29 (0.87–6.07)
Very or quite concerned effect COVID-19 on child	0.89 (0.62–1.29)	0.43 * (0.20–0.94)
High risk/moderately concerned risk contracting COVID-19 child	1.28 (0.89–1.82)	3.40 ** (1.59–7.28)
Household member self-isolated	0.99 (0.67–1.48)	0.72 (0.34–1.52)
Easy access to GP	0.68 * (0.47–0.98)	0.84 (0.41–1.74)
Easy access to ED	0.97 (0.67–1.40)	1.21 (0.60–2.44)

Note: * *p* < 0.05, ** *p* < 0.01.

## Data Availability

The datasets used and analysed during the current study have been submitted to the Irish Social Science Data Archive (ISSDA).

## Appendix A

**Table A1:** Survey Questions.

Survey	
1. What is your gender?	- Male - Female - Other - Prefer not to say
2. What age are you?	- <20 - 20–29 - 30–39 - 39–49 - 50 or over
3. How many children do you have in these age groups?	- Under 2 - 2–4 - 5–9 - 10–16
4. What county do you reside in?	[drop-down option]
5. What is your marital status?	- Single - Married - Cohabiting - Widowed - Divorced/Separated
5a Display If single or divorced/separated: Are you a one-parent family or co-parent?	- One parent - Co-parent
6. When making decisions about healthcare for your child or children under 16, do you discuss with other people?	- Yes (Please describe who) - No
7. Do you hold a medical card?	- Yes - No
8. Do you hold a GP visit card?	- Yes - No
9. Does your family have private medical insurance?	- Yes - No
10. Are you a healthcare professional?	- Yes - No
11. What is your highest level of education?	- Lower Secondary Education - Upper Secondary Education - Post-secondary certificate or vocational education - Degree or postgraduate third level education
12. Does anyone in your household have any of the following conditions/illnesses? Indicate if it is one of your children under 16, yourself or another household member, or none	- Currently undergoing cancer treatment or recently covered from cancer - A chronic lung condition (e.g., asthma) - Diabetes - High blood pressure/hypertension - A condition, or are taking medication, that means you are immunocompromised - An Intellectual disability - Heart, kidney, or liver disease or organ transplant
12a Display if a child under 16 is selected: Please answer the following questions thinking about the child who is under 16 with a health condition. If you have more than one child who has a health condition, please answer with one child in mind.	- Age of child [AGE] - Approximately how many times in the last year has your child needed the following healthcare: - A visit to the GP [NUMBER] - A visit to the Emergency Department [NUMBER] - An outpatient appointment (e.g., visiting a specialist) [NUMBER] - An overnight stays at hospital [NUMBER]
13. Since the schools closed and the “lockdown” was announced, have you needed to access any healthcare for your child or children under 16?	- Yes, we accessed healthcare - Yes, but we didn’t access healthcare - No, we didn’t need to
13a Display if yes, we accessed healthcare Where did you seek healthcare?	- We attended the GP in person - We had a consultation with GP by phone/video - We attended the ED in person - I used a service provided by my health insurance in person - I used a service provided by my health insurance over the phone/video - I used another service [PLEASE SPECIFY]
13b Display if yes, but we didn’t access healthcare Why did you decide to not access healthcare? You can choose more than one option	- I was worried about catching COVID-19 - I was worried about being judged for using a service if it wasn’t an emergency - I thought the service would be needed more urgently by other people - I thought the government advice meant I couldn’t go to the GP or hospital - I was worried that the service would be extremely busy and that I would have to wait for too long - I don’t have a car and didn’t want to use public transport - Something else (please explain)
13c Display if yes accessed healthcare is selected When you decided to seek healthcare, was any of the following a concern for you?	- I was worried about catching COVID-19 - I was worried about being judged for using a service if it wasn’t an emergency - I thought the service would be needed more urgently by other people - I thought the government advice meant I couldn’t go to the GP or hospital - I was worried that the service would be extremely busy and that I would have to wait for too long - I don’t have a car and didn’t want to use public transport - Something else (please explain)
13d Display if no we didn’t need to access healthcare If you needed to access healthcare for your child, would any of the following a concern for you?	- catching COVID-19 - being judged for using a service if it wasn’t an emergency - the service would be needed more urgently by other people - the government advice meant I couldn’t go to the GP or hospital - the service would be extremely busy and that I would have to wait for too long - I don’t have a car and didn’t want to use public transport - Something else (please explain)
14. When the schools closed and the lockdown was announced, would you say you felt more hesitant to seek healthcare for your child or children under 16?	- Much more - Somewhat more - About the same - Somewhat less - Much less
15. Now that we are getting used to the COVID-19 restrictions, do you feel more or less hesitant about accessing healthcare for your child or children under 16?	- Much more - Somewhat more - About the same - Somewhat less - Much less
16. Which of these sources have you seen or used for advice on when to use health services since the COVID-19 restrictions have been in place?	- Government sources (such as on TV or radio, distributed leaflets, briefings from health officials or government) - HSE sources (such as HSE website, mychild.ie or your GP’s website) - Other expert online sources (such as university or other organisations) - Experts on Social Media sites (such as Twitter, Facebook) - Family/friends on Social Media sites - Other - None of the above
17. Have you had to self-isolate due to COVID-19?	- Yes - No
17a Display if answered yes Please indicate why	- Cocooning due to chronic illness - In contact with someone with suspected or confirmed COVID-19 - Suspected Covid-19 - Confirmed Covid-19
17b Display if confirmed COVID-19 is selected Were you hospitalised as a result?	- Yes - No
18. Do you worry that your child or children under 16 is at risk of contracting COVID-19?	- not at all - slightly concerned - moderately concerned - high risk
19. Do you feel if your child or children under 16 contracted COVID-19 that they will be:	- unaffected - somewhat unwell - quite sick - very unwell
20. Has a member of your household had to self-isolate due to COVID-19?	- Yes - No
20a Display if yes is selected Please indicate why:	- Cocooning due to chronic illness - In contact with someone with suspected or confirmed COVID-19 - Suspected Covid-19 - Confirmed Covid-19
20b Display if confirmed with COVI9-19 was selected Were they hospitalised as a result?	- Yes - No
21. Has someone you know died because of COVID-19?	- Yes - No If you or someone you know has directly experienced COVID-19, please provide further information of this below.
22. Taking into account distance, transport and appointment availability, how easy is it for you to access your GP on behalf of your child/children:	- very easy - usually easy - I can if I need to - somewhat difficult - very difficult
23. Taking into account distance and transport, how easy is it for you to access your nearest Emergency Department that looks after children:	- very easy - usually easy - I can if I need to - somewhat difficult - very difficult
24. Is it easier for you to access a Local Injury Unit or Urgent Care Centre for emergency care for your child/children than the ED?	- Yes - no - not sure where my LIU or UCC is located
25. Stress Scale of the Depression, Anxiety, and Stress Scale (DASS 21; Lovibond and Lovibond, 1995)	
